# Health service engagement, side effects and concerns among men with anabolic-androgenic steroid use: a cross-sectional Norwegian study

**DOI:** 10.1186/s13011-023-00528-z

**Published:** 2023-04-03

**Authors:** Hans Christian Bordado Henriksen, Ingrid Amalia Havnes, Marie Lindvik Jørstad, Astrid Bjørnebekk

**Affiliations:** 1 Anabolic Androgenic Steroid Research Group, Section for Clinical Addiction Research, Division of Mental Health and Addiction, Oslo University Hospital, Oslo, Norway; 2 Institute of Clinical Medicine, University of Oslo, Oslo, Norway; 3 Division of Mental Health and Addiction, Oslo University Hospital, Oslo, Norway; 4 National Advisory Unit on Substance Use Treatment, Oslo University Hospital, Oslo, Norway

**Keywords:** Anabolic-androgenic steroids, Image and performance enhancing drugs, Treatment-seeking behaviour, Health service engagement, Physical health, Mental health, Health concerns, Side effects

## Abstract

**Background:**

Recreational use of anabolic-androgenic steroids (AAS) is a public health concern world-wide associated with a range of physical and psychological side effects. Still, people who use AAS tend to be reluctant to seek treatment. This study aims to explore use characteristics, treatment-seeking behaviour, side effects and associated health concerns among men with AAS use.

**Methods:**

The study includes cross-sectional self-report data from 90 men with a current or previous use of AAS exceeding 12 months, where 41 (45.6%) had sought treatment at least once during their lifetime, and 49 (54.4%) had not. Health service engagement was examined with descriptive statistics on reasons for contacting health services, transparency about AAS use, satisfaction with health services and reasons for not seeking treatment. Furthermore, experienced side effects and health concerns were compared between the *treatment seeking* and the *non-treatment seeking* group, using two-sample t-tests and Chi^2^ or Fisher exact tests for numerical and categorical variables, respectively.

**Results:**

All 90 AAS-using men reported side effects from AAS use. Treatment seekers were significantly younger, experienced more side effects including gynecomastia, excessive sweating, fatigue, depression and anxiety, and expressed more concern for testosterone deficiency. Preventive health check-up was the most common reason for seeking treatment (n = 22, 53.7%), and 38 men (93%) were transparent about AAS use during consultations with health professionals. The main reported reasons for not seeking healthcare services were that the experienced side effects were not considered to be of treatment demanding nature (n = 39, 79.6%) and the belief that healthcare providers had scarce knowledge about AAS use and its health impacts (n = 12, 24.5%).

**Conclusions:**

Reluctance to seek treatment among people who use AAS, despite having associated side effects and health concerns, may contribute to continued health risks. It is important to fill the knowledge gap on how to reach and treat this new patient group, and policy makers and treatment providers need to be educated on how to meet their treatment needs.

## Background

Anabolic-androgenic steroids (AAS) constitute the male sex hormone testosterone, as well as manufactured synthetic androgens with similar effect and structure [1]. Non-prescribed AAS have been used among professional elite athletes to enhance performance since the 1950s, but is today increasingly more common among recreational gym-goers who use them to improve body image, build muscles more easily and increase the feeling of well-being [2–4]. A meta-regression analysis have suggested that lifetime prevalence rates among men varies between 4 and 6% [2].

### Adverse effects from AAS use

AAS use is associated with several side effects, where the extent and severity of health issues seems to depend on AAS type, dose, accumulated time of use, method of administration and unknown predisposing factors [5, 6]. Adverse health effects include cardiovascular harm, infertility, gynecomastia, neurocognitive impairment and musculoskeletal damage [5, 7–12]. In addition, about 30–50% of people who use AAS develop dependence, characterized by continued AAS use despite adverse physical and mental health effects [13]. The dependence is highly linked to symptoms such as depression, fatigue and sexual dysfunction, which are commonly experienced during AAS cessation due to low endogenous levels of testosterone [14–19].

### Health service engagement among people who use AAS

Despite the likely risk of use-related health implications and associated health concerns, people who use AAS often show reluctance to contact health services. A new meta-analysis estimated that only 37% of individuals using AAS seek support from physicians [20]. In addition, Zahnow et al. (2017) showed that 67% do not engage health services despite having AAS-associated health concerns [21]. Among those seeking support from clinicians, many do not disclose their AAS use [21–23]. Frequently reported reasons for not seeking treatment or not disclosing use are beliefs that physicians lack knowledge about AAS [23], or are not able to help [24]. Other reasons include concerns about stigma [25] or that the problem is not significant enough to seek help [21, 26–28]. Many prefer to seek information from peers [29, 30] and online sources [31–33] on how to avoid and handle side effects [34]. However, in Norway, an anonymous national information service on AAS use, its side effects and the treatment options available, have been helpful for some [35]. As use of AAS is still a young form of substance use that first became prevalent among the general population in the late 80s [36], we might expect an increase of patients with health problems related to long-term AAS use in the coming decades [5]. It is possible that reluctance to seeking treatment will constitute a higher continued health risk. To tailor health services to people who use AAS, knowledge on what kind of support they want and what they find useful is essential [29, 37]. In addition, knowledge is needed about AAS-induced side effects and the health service engagement in relation to these. Some studies have explored experiences with customised harm reduction services [30, 38] and endocrine outpatient clinics for people who use AAS [18, 39]. Other studies have included international samples [21, 37, 40] with great variations in how public and private health services are organized and paid for, meaning that study participants in these studies may not have access to similar health services. Hence, there is still a knowledge gap regarding treatment-seeking experiences and tailored treatment options for this heterogeneous patient group [41]. Norway constitutes a particular setting as people with current or previous AAS use have rights to substance use disorder (SUD) treatment, and public health services are widely available. It may therefore be useful to gather more information about health service engagement, AAS-related health concerns and side effects among people in Norway with current or previous use of AAS.

### Aims

In a sample of Norwegian men with current or previous AAS use, we explored characteristics of treatment-seeking behaviour. We wanted to investigate health service engagement related to AAS use, whether those who seek health services during their lifetime experience more side effects or health concerns compared to those who do not, rationales for seeking treatment, experiences with the healthcare system, and contributing factors for not engaging health services.

## Methods

### Setting

In Norway, all inhabitants are appointed a publicly funded General Practitioner (GP), who can refer their patients on indication to specialized health care facilities. However, patients may also choose to bypass their GP and seek treatment directly in private health care facilities at their own expense. In 2012, persons with current or previous AAS use and related health problems were given rights to outpatient specialized SUD-treatment. The treatment involves psychosocial support of persons with current or previous AAS use, follow-up on withdrawal symptoms commonly experienced during or after AAS cessation, and treatment of health problems related to current or previous use. In 2013, the Norwegian Drug Act was modified, and use and possession of AAS and other image and performance enhancement drugs became illegal.

### Study design

The study is part of a longitudinal study investigating the impact of high-dose AAS use on the brain health and behavior at Oslo University Hospital in Norway [42–44]. The present paper utilizes cross-sectional data collected with self-report web questionnaires in 2017–2019 where a national information service for AAS inspired variables linked to health concerns, treatment-seeking behaviour and treatment satisfaction for people with current and previous AAS use [35], together with elements from Zahnow et al. (2017) [21] adapted for a Norwegian context. The self-experienced side effects are meant to cover a broad spectrum of medical and psychological side effects that high-dose AAS use may cause, based upon literature in the field [5, 45], whereas some (e.g. cognitive side effects) are adapted for the purpose of the longitudinal study. Social media and other relevant online forums were used as recruitment tools, and study posters and flyers were shared at various gyms in Oslo. In total, 90 males above 18 years of age with capacity of giving consent and with a cumulative AAS use of at least 1 year were included.

### Measures and variables

#### Demographic factors

Demographics included age, education, work status and parenthood, as well as alcohol consumption and use of prescribed psychopharmacological medication (i.e. antidepressants, anxiolytics or antipsychotics). Variables for AAS use characteristics were debut age, current or previous use, accumulated use in years, side effects from use and dependence.

#### AAS-dependence

Dependence was evaluated using a version of the Structured Clinical Interview for DSM-IV (SCID II) [46] based upon the standard substance-dependence criteria of DSM–IV and adapted to apply to AAS-dependence [24, 47]. The adapted SCID comprises seven items: tolerance, withdrawal, substance used more than intended, desire to cut down on use, much time using substance or recovering from effects, important activities given up due to use and continued use despite health problems. AAS dependence was defined as three or more items coded as severe and occurring during the same 12-month period [47].

#### Substance use and psychopharmacological treatment

Alcohol consumption was evaluated by a self-reported version of the 10-item screening test “The Alcohol Use Disorders Identification Test” (AUDIT), where a score of 8 or more depicts harmful alcohol use [48, 49]. Illicit non-prescribed use and drug-related problems were evaluated using the Drug Use Disorders Identification Test (DUDIT), a screening tool for harmful substance use and dependence following the ICD-10 and DSM-IV diagnostic systems [50, 51]. A score of 6 or more on DUDIT depicts harmful use among men. Regular illicit substance use was defined as use of substances other than alcohol on a monthly basis. Psychopharmacological treatment included self-reported prescribed current or lifetime use of either antidepressants, anxiolytics or antipsychotics.

#### AAS-related side effects

Mental health was assessed by questions addressing to what degree they had experienced psychological side effects, with options ranging from not experienced to a mild, moderate or severe degree. The psychological effects included: Fatigue, sleep disorder, depression, mood swings, anxiety, paranoia, irritability, short fuse, aggression, jealousy, increased impulsivity, reduced empathy, and reduced memory and/or concentration. For analysis purposes, the participants’ answers were divided into dichotomous variables (yes/no) depending on whether they experienced psychological symptoms or not. For the physical side effects, the participants answered “yes” or “no” to having experienced one or more of the following: stretch marks, acne, excessive sweating, oedema, hair growth, hair loss, ruptured tendons or muscles, sore injection sites, abscess, gynecomastia, reduced libido, sexual dysfunction and testicular atrophy.

#### Health concerns

The participants were asked to rate their level of concern for common AAS-related side effects, with the options “not worried”, “a little worried” or “very worried”. For analysis purposes, “a little worried” and “very worried” were grouped together to a “worried”-group and concerns were analysed as dichotomous variables (yes/no). The different health concerns for various AAS-related health problems are listed in Table 3.

#### Health service engagement

Contact with health services during lifetime due to AAS-related side effects or health concerns was measured by contact vs. no contact. If contact had been made, a follow-up question was given with information on which health service had been sought: assigned public GP, a public specialist through a referral from their GP (i.e. a SUD department or specialized somatic health care) or private health care (i.e. private general practitioner and/or a specialist in various somatic health disciplines). Hence, the term ‘health service’ used in this paper refers to any of the above mentioned healthcare services.

#### Reasons for seeking treatment

The participants were asked to tick off one or more AAS use-related reasons for seeking treatment including preventive health check-ups, mental health issues (depression, anxiety, mood swings, irritability, anger, aggression, suspiciousness, and/or other), impaired cognitive function (reduced memory and/or concentration), cosmetic complaints related to skin and hair (acne, stretch marks, pattern hair loss and/or hair growth), sexual dysfunction, change of sex organs (testicular atrophy), concerns with internal organs (heart, liver and/or kidneys), gynecomastia, musculoskeletal harm and testosterone deficiency (hypogonadism). In addition, other reasons for health service engagement could be specified in an open response.

#### Disclosure of AAS use

The transparency about use in meetings with physicians was measured by answering “yes, the physician brought it to light, but I did not disclose use”, “yes, the physician brought it up”, “yes, I brought it up myself” or “no, the physician did not bring it up and I did not wish to disclose use.”

#### Health service experience and satisfaction levels

The participants were asked whether they experienced healthcare workers to have enough knowledge about AAS (yes/no/other). Participants’ experiences with the health services were rated with scales from 1 (worst) to 5 (best). For analysis purposes, 1 (very dissatisfied) and 2 (a little dissatisfied) were combined as ‘dissatisfied’, and 4 (a little satisfied) and 5 (very satisfied) were combined as ‘satisfied’.

#### Reasons for not seeking treatment

Participants who reported not to have engaged health services were given the following response options for not seeking treatment: feeling of shame, fear of stigma, a belief that the physician could not and/or did not wish to provide treatment, a perceived low knowledge on AAS and its health consequences among health professionals, not experiencing side effects to be serious enough, preferring to self-medicate to prevent or treat AAS-related side effects, fear of legal repercussions (i.e. police fines, child protection services, employer and/or driving license services) and economic reasons (i.e. costs for consultations). The participants could choose one or more options and had the possibility to specify other reasons in an open response.

### Statistical analyses

Two-sample t-tests were used for between-group comparisons of numerical variables, while Chi^2^-tests and Fisher’s exact tests were applied to compare categorical and dichotomous variables. A two-sided p-value of ≤ 0.05 was considered statistically significant. All statistical calculations and analyses were performed using STATA (version 17.0, StataCorp LLC, Texas, USA). There were no missing data from the online questionnaires as mandatory answers were used.

### Ethics

All research has been carried out in congruence with the Declaration of Helsinki. The Regional Committees for Medical and Health Research Ethics South East Norway (REC) (2013/601) approved the study prior to data collection. Written consent was collected from all participants at the time of inclusion. A total amount of NOK 500 (**≈**$50) was given as compensation for taking part in the main research project. All participants had the opportunity to discontinue the study at any point.

## Results

### Demographics and characteristics of AAS use

Demographic data and AAS use characteristics are presented in Table 1. Those seeking treatment were 4.9 years younger (mean age = 36.3 years, SD = 11.3) than the non-treatment seeking men (41.2 years, 9.5), p = 0.029 and had a higher current use of prescribed anxiolytics at the time of inclusion (n = 8, 19.5% vs. n = 1, 2%), p = 0.006. There were no significant differences between treatment and non-treatment seekers regarding AAS debut age, years of accumulated use or proportion of current use.

**Table 1:** Demographics and characteristics related to AAS use

	Whole sample (*n *= 90)		Treatment seeking group (*n* = 41)		Non-treatment seeking group (*n* = 49)			
	Mean (SD)		Mean (SD)	Range	Mean (SD)	Range	t	p-value
Age (years)	38.9 (10.6)		36.3 (11.3)	22-74	41.2 (9.5)	25-60	2.216	0.029*
Education (years)	14.8 (2.4)22		14.8 (2.3)	9-19	14.8 (2.6)	10-23	-0.072	0.943
AAS debut age	22.6 (7.8)		21.6 (7.8)	15-55	23.5 (7.9)	16-50	1.161	0.249
Accumulated AAS use (years)	11.9 (8.5)		11.6 (8.7)	1.5-29	12.2 (8.4)	1-29	0.338	0.736
	*n*	%	*n*	%	*n*	%	χ2	p-value
AAS dependence (SCID, 5 missing)	52 (n = 85)	61.2	24 (n = 40)	60.0	28 (n = 45)	62.2	0.044	0.834
Current AAS use	59	65.6	25	61.0	34	69.4	0.7	0.403
Current employment	70	77.7	31	75.6	39	79.6	0.205	0.651
Student	15	16.7	6	14.6	9	18.4	0.224	0.636
Children	57	63.3	26	63.4	31	63.3	0.002	0.988
AU/week (mean, SD, range, t)	2.2 (2.9)	0-12	2.4 (3.3)	0-12	1.9 (2.6)	0-10	0.797	0.428
AUDIT ≥ 8	17	19.5	6	15.4	11	22.9	0.776	0.378
Regular illicit substance use	26	28.9	10	24.4	16	32.7	0.742	0.389
DUDIT ≥ 6	12	13.3	7	17.1	5	10.2	0.912	0.340
Psychopharmacological treatment, life-time	36	40	19	46.3	17	34.7	1.262	0.261
Psychopharmacological treatment, current	13	14.4	8	19.2	5	10.2	1.565	0.211
Antidepressants, life-time	21	23.3	13	31.7	8	16.3	3.195	0.074
Antidepressants, current	5	5.5	2	4.9	3	6.1	0.066	1.000
Anxiolytics, life-time	23	25.5	12	29.3	11	22.4	0.546	0.460
Anxiolytics, current	9	10	8	19.5	1	2.0	7.571	0.010*
Antipsychotics, life-time	5	5.5	3	7.3	2	4.1	0.452	0.656
Antipsychotics, current	4	4.4	2	4.9	2	4.1	0.033	1.000

Data are presented as means (standard deviation, SD) and n (%). *Significant difference between the groups (p ≤ 0.05). Fischer’s exact test was used when the expected number were based upon less than five cases. AAS = Anabolic-androgenic steroids, SCID = Structured Clinical Interview for DSM-IV, SD = standard deviation, AU = Alcohol units, AUDIT = Alcohol Use Disorders Identification Test, DUDIT = Drug Use Disorders Identification Test.

### Physical and psychological side effects

All of the 90 AAS-using men in the study reported one or more side effects from use, where 41 (45.6%) men had ever sought treatment due to AAS-related side effects or associated health concerns, and 49 (54.4%) had not. The mean of total experienced side effects were 15.7 (SD 6.3, range 4–28) for the treatment-seeking group and 13.1 (6.3, 2–28) for the non-treatment seeking group, p = 0.049, whereas the numbers for physical side effects were 8.8 (SD 3.5, range 2–15) vs. 7.5 (SD 3.7, range 1–15), p = 0.106, and for psychological side effects 6.8 (4.2, 0–14) vs. 5.5 (3.7, 0–13), p = 0.123. The individual self-reported psychological and physical side effects are presented in Table 2. Fatigue (63.4% vs. 34.7%, p = 0.007), depression (48.8% vs. 26.5%, p = 0.029), anxiety (43.9% vs. 22.4%, p = 0.030), gynecomastia (48.8% vs. 24.5%, p = 0.017) and excessive sweating (80.5% vs. 57.1%, p = 0.018) were significantly more reported in the treatment-seeking group.

**Table 2:** Side effects from AAS use

	Whole sample (n = 90)			Treatment group (*n* = 41)		Non-treatment group (*n* = 49)				
**Self-reported AAS-related psychological side effects**	*n*		*%*	*n*	%	n	%	χ2	p-value	
Fatigue	43		47.8	26	63.4	17	34.7	7.38	0.007*	
Sleep disorder	59		33.3	30	73.2	29	50.8	1.934	0.164	
Depression	33		36.7	20	48.8	13	26.5	4.759	0.029*	
Mood swings	61		67.8	29	70.7	32	65.3	0.301	0.583	
Anxiety	29		32.2	18	43.9	11	22.4	4.704	0.030*	
Paranoia	13		14.4	9	22.0	4	8.2	3.434	0.077	
Irritability	54		60.0	23	56.1	31	63.3	0.478	0.489	
Short fuse	55		61.1	25	61.0	30	61.2	0.001	0.981	
Aggression	38		42.2	18	43.9	20	40.8	0.087	0.768	
Jealousy	60		66.7	24	58.5	36	73.5	2.234	0.134	
Increased impulsivity	38		42.2	15	36.6	23	46.9	0.981	0.322	
Reduced empathy	34		37.8	15	36.6	19	38.8	0.046	0.831	
Reduced memory	58		64.4	24	58.5	34	69.4	1.147	0.284	
Reduced concentration	32		35.6	18	43.9	14	28.6	2.29	0.130	
**Self-reported AAS-related physical side effects**	*n*		*%*	*n*	%	*n*	%	χ2	p-value	
Stretch marks	46		51.1	23	56.1	23	46.9	0.749	0.387	
Acne	59		63.3	27	65.9	32	65.3	0.003	0.957	
Excessive sweating	61		67.8	33	80.5	28	57.1	5.57	0.018*	
Oedema	63		70.0	29	70.7	34	69.4	0.019	0.890	
Hair growth	46		51.1	25	61.0	21	42.9	2.933	0.087	
Hair loss	29		32.2	10	24.4	19	38.8	2.115	0.146	
Ruptured muscles or tendons	24		26.6	12	29.3	12	24.5	0.261	0.610	
Injection site pain	72		80.0	34	82.9	38	77.6	0.403	0.525	
Abscess	23		25.6	12	29.3	11	22.4	0.546	0.460	
Gynecomastia	32		35.6	20	48.8	12	24.5	5.748	0.017*	
Reduced libido	30		33.3	16	39.0	14	28.6	1.098	0.295	
Sexual dysfunction	25		27.8	14	34.1	11	22.4	1.523	0.217	
Testicular atrophy	63		70.0	31	75.6	32	65.3	1.129	0.288	

Data are presented as numbers (n) and percentages (%). *Significant difference between the groups (p ≤ 0.05). Fischer’s exact test was used when the expected number were based upon less than five cases.

### Concerns about AAS-related health issues

Concerns about AAS-related health effects were generally more common among men who sought treatment, where 73% (n = 30) reported having concerns, compared to 53% (n = 26) of those who did not seek health services (p = 0.050). The different health concerns for various AAS-related health problems are listed in Table 3.

**Table 3:** Health concerns on side effects from AAS use

		Whole sample (n = 90)		Treatment seeking group (*n* = 41)		Non-treatment seeking group (*n* = 49)		*χ2*	*p-value*
		*n*	*%*	*n*	*%*	*n*	*%*		
Having AAS-related health concerns		56	62.2	30	73.2	26	53.1	3.840	0.050*
Internal organs		48	53.3	26	63.4	22	44.8	3.075	0.080
Testosterone deficiency		40	44.4	25	61.0	15	30.6	8.335	0.004*
Mental health		32	35.6	16	39.0	16	32.7	0.396	0.529
Sexual dysfunction		29	32.2	14	34.1	15	30.6	0.128	0.721
Gynecomastia		26	26	14	34.1	12	24.5	1.013	0.314
Skin and hair		25	28.9	14	34.1	11	22.4	1.523	0.217
Cognitive function		19	21.1	10	24.4	9	18.4	0.486	0.486
Musculoskeletal harm		8	8.9	4	9.8	4	8.2	0.070	1.000

Data are presented as numbers (n) and percentages (%). *Significant difference between the groups (p ≤ 0.05). Fischer’s exact test was used when the expected number were based upon less than five cases.

### Reasons for seeking treatment

The most common reported reasons to seek treatment, as presented in Table 4, were preventive health check-ups, mental health issues, testosterone deficiency, problems related to internal organs and sexual dysfunction. Other reasons not mentioned in Table 4 included musculoskeletal harm (1), high haematocrit levels (1) and skin abscess requiring antibiotics (1).

**Table 4:** Reasons for seeking treatment (n = 41)

	*n*	%
Preventive health check-up	22	53.7
Mental health	12	29.3
Testosterone deficiency	11	26.8
Internal organs	11	26.8
Sexual dysfunction	8	19.5
Skin and hair	5	12.2
Gynecomastia	5	12.2
Other	3	7.3
Testicular atrophy	3	7.3
Cognitive function	2	4.9

Given reasons for engaging health services (several responses possible) are presented as numbers (n) and percentages (%).

### Experiences with health services for AAS-related health problems

**Fig. 1:**
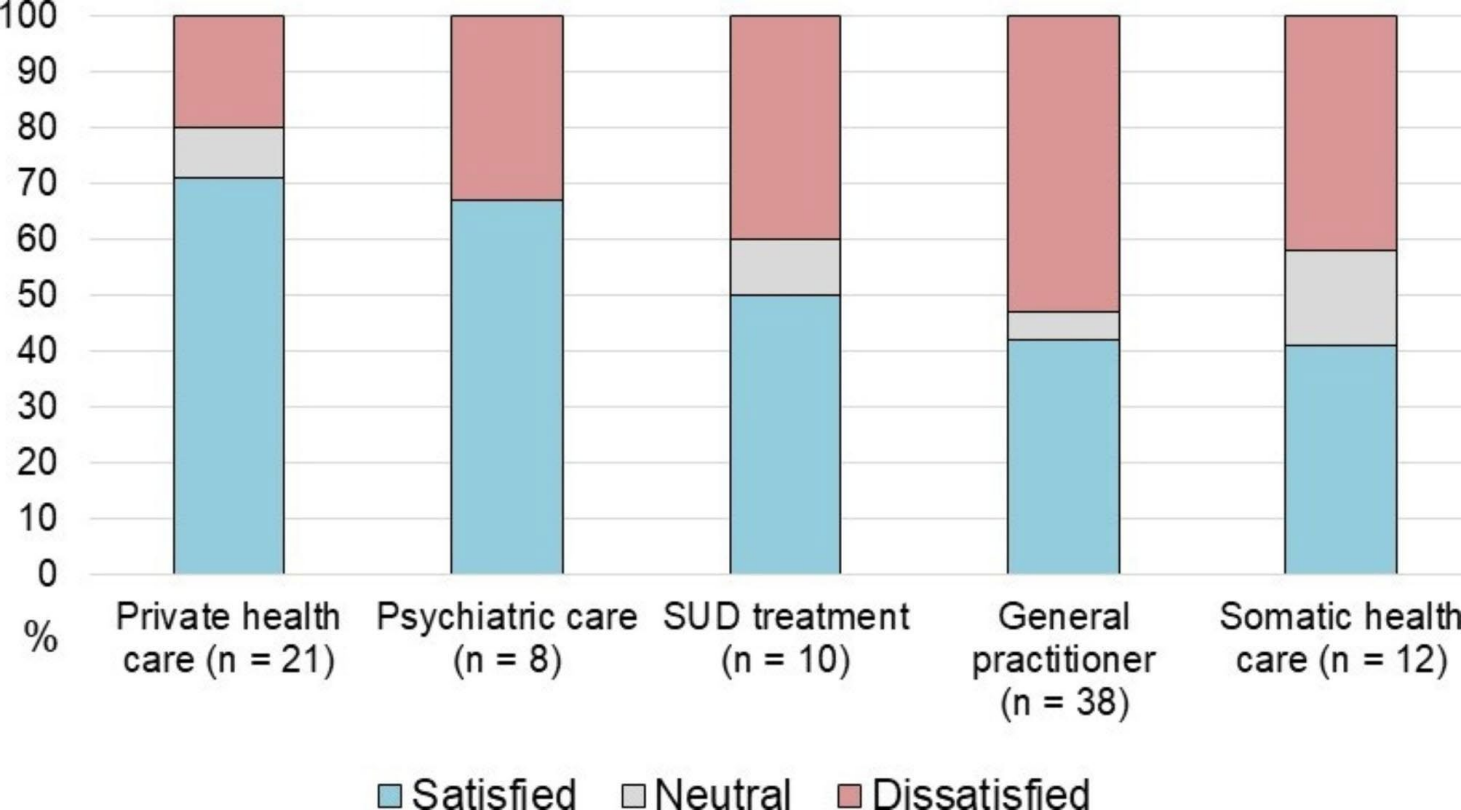
Number of participants who visited the different health services and associated satisfaction level for each service. Data are presented as percentages of satisfied, neutral or dissatisfied of the total number

The number of treatment seekers for each health service and the associated satisfaction levels are shown in Fig. 1. 78% (n = 32) of those seeking treatment had contact with a single health service, 15% (6) had contact with two and 7% (3) had contact with three health services (i.e. their general practitioner, a publicly funded specialist *and* private health care). Seven out of eleven participants who sought help for testosterone deficiency, reported to have visited a private health service. During consultations, 88% (36) of treatment seekers disclosed AAS use themselves, whilst 5% (2) shared information about use when physicians brought it up. The use was not brought up at all in 7% (3) of the cases. While 51% (21) believed that the physicians and other healthcare personnel had enough knowledge about AAS, 41% (17) did not.

### Reasons for not seeking treatment

Overall, 49 participants had not sought health services for AAS-related issues. The main given reason for not seeking treatment was not experiencing side effects to be of treatment-demanding nature (n = 39, 79.6%). Twelve participants (24.5%) considered their physicians to have little knowledge about AAS, nine (18.4%) preferred to self-medicate and six (12.2%) did not believe that the health services could offer useful treatment. Other reasons for not seeking treatment included fear of legal repercussions (4), fear of judgement or stigma from health professionals (3), doubting physicians’ wish to help (3) or feeling too ashamed (2). No other reason for not seeking treatment was reported.

#### Discussion

AAS use is associated with physical and psychological side effects that often generate health concerns among those who use. In a sample of 90 men with current and former AAS use, we investigated lifetime treatment-seeking behaviour, experienced side effects from use and associated health concerns. Less than half of the men (n = 41, 45.6%) had been in contact with health services. These men were younger, experienced somewhat more side effects from use and had more AAS-related health concerns compared to the men who had not sought treatment. Preventive health check-up was reported as the main reason for engaging health services, while not regarding the experienced side effects to be serious enough was the most common reason not to.

Despite the fact that the proportion who contacted health services in our sample might be considered low, it is found even lower in other studies [20, 21]. A recent systematic review and meta-analysis estimated the prevalence of seeking support from physicians to be 37% among those who use AAS, but with large variations depending on age, access to needle and syringe exchange programs, and geographical locations [20]. Interestingly, the highest health service engagement was seen in Australia (67%). Australia and Norway have similar public information services that have educated physicians on how to monitor and follow up on AAS use [35, 52], which might have positively influenced the treatment-seeking behaviour.

Similarly to previous studies, the most common reasons for not seeking professional help was not considering the side effects to be of treatment demanding nature [23, 26, 27], or the belief that physicians lacked knowledge on the topic of AAS [21–23, 40]. As some physicians might not have adequate knowledge on AAS or routines of mapping use among their patients [23, 53, 54], people who use AAS tend to seek information from each other or elsewhere, in settings which is often referred to as “bro-science” in different online drug communities [55]. The bro-science culture has been perceived as a safe online environment for sharing AAS expertise, exercise and diet advice, as well as recommendations on physical and mental health self-monitoring [31, 56, 57]. Many consider this self-research and trial-and-error practice more trustworthy than taking advice from healthcare workers [29]. AAS-related unspecific health examinations used as preventive measures have previously shown to affect health service engagement positively [16, 21]. In fact, preventive health check-ups was reported as the most common reason for seeking health services in our sample. However, the health gain of preventive measures in asymptomatic persons who use AAS remains little studied. In contrast to previous studies [21, 35], treatment seekers in our study were younger of age. It is possible that younger people who use AAS are more concerned about their health. As AAS use is linked to potential severe health risks such as cardiovascular disease including sudden cardiac death [58], this possible new trend of younger and seemingly more concerned treatment seekers might work in favour in preventing future serious adverse effects.

Testosterone deficiency due to AAS-induced hypogonadism (ASIH) is a common side effect from AAS use, characterized by fatigue and depressive symptoms, and known to impair both libido and sexual function [14]. Symptoms of ASIH usually become apparent during or after AAS cessation [14], contributing to AAS restart among many [24, 29, 47]. In most cases, the hypothalamic-pituitary-testicular (HPG) axis recovers within 3–18 months, although some experience reduced endogenous testosterone levels with associated symptoms years after withdrawal [59]. In the current study, concerns for testosterone deficiency were dominant among treatment seekers. Still, only 27% reported this as a reason for seeking treatment, which could be explained by the lack of clinical guidelines on how to treat ASIH. Testosterone substitution or post cycle therapy (PCT), a treatment to enhance endogenous testosterone levels after AAS cessation [60], is generally not recommended during AAS withdrawal [17, 61], as HPG impairment tends to be only temporary. In addition, physicians are also often unwilling to prescribe such treatment, not only due to the lack of research on the area but also to AAS’ illegal status in the country [62]. However, non-prescribed PCT is common among people who use AAS [39, 63], and they seek information online and among peers [29, 32–34]. This might be part of the reason why so few seek health services due to ASIH following AAS cessation. Although PCT may reduce symptoms of ASIH, mental health problems seem to be harder to alleviate. In fact, depression, anxiety and fatigue were all side effects more commonly reported among those who sought treatment, and mental health issues was the second most common reason for seeking health services, a finding that is in concordance with previous studies [21, 35]. AAS use has recently shown to be associated with increased use of prescribed psychopharmacological treatment including anxiolytics [64], which was also reflected in the present study as treatment seekers reported significantly more current use of prescribed anxiolytics compared to the non-treatment seeking group. Moreover, it is likely that predefined perceptions of which treatment each health service can provide will influence health service engagement [27]. This could explain why a high proportion of men who sought help for testosterone deficiency due to ASIH chose to engage private health care clinics. The high satisfaction levels with private health care might reflect easier access to clinical examinations, testosterone substitution or PCT, including less reporting practises in private clinics. At the same time, mental health was the main motivating factor for seeking SUD treatment, as this therapeutic approach focuses mainly on psychosocial interventions and symptom-relieving treatment, targeting conditions such as depression, anxiety and sleep disorder [28].

Previous studies have addressed how a high proportion of people who use AAS do not seek treatment despite experiencing AAS-related side effects and health concerns [21, 22, 27, 65, 66]. More than half of the sample in the present study had never sought treatment, despite having an average duration of AAS use that exceeded ten years, 53% having current health concerns and 62% fulfilling the criteria for AAS dependence. In addition, all of the *non*-treatment seekers reported having experienced adverse effects from use with an average of 13 different side effects. For instance, more than 60% of men from this group reported side effects such as reduced memory, mood swings, acne, oedema and testicular atrophy. As long-term use is associated with multiple and even more severe health risks [5, 7, 8, 11, 14–16], it is likely that underlying pathology in these individuals remains undiagnosed and untreated.

Previous studies have suggested that more AAS knowledge among healthcare workers is needed to make treatment-seeking more appealing and less stigmatizing [67]. Since 2014, the National Steroid Project through Oslo University Hospital in Norway has systematically spread information to the Norwegian public on AAS use through social media [68] as well as educated health professionals on how to treat AAS-related health issues [35]. In addition, as one of few countries, people who use AAS in Norway have rights to specialized SUD treatment in the public health care system. These factors could have contributed to a higher percentage of health service engagement in our sample compared to other studies, but also to the notable transparency about AAS use, as disclosure was reported by 93% of the treatment seekers during health care visits. These encouraging findings differ considerably from previous studies [21–23], even though 24% of those who disclosed use were involved in SUD treatment, meaning that transparency about AAS use was likely a precondition for starting treatment.

#### Limitations

Our study involved self-report questionnaires that could be susceptible for exaggerations, social desirability-bias and missing data through incomplete surveys, although the latter was reduced by using online questionnaires with mandatory answers. The answers might also be subject to recall and reporting bias if use ended years ago, or if health care visits were far back in time. Reported health concerns in our study gives a picture of current worries about adverse events, even though use may have ended several years ago. The study shows that measurement of lifetime treatment-seeking related to adverse effects of AAS use might be an important measure for people who use AAS, especially among those who never engage health services.

## Conclusion

In our study, treatment-seeking behaviour among men who use AAS was associated with age, health concerns, experienced side effects from use and how serious these effects were perceived. Even though long-term AAS use is likely to have a negative impact on health, most of those who use tend to avoid health services. It is likely that such reluctance to treatment-seeking will constitute a higher continued health risk. Any health service may be a potential starting point to assess and treat side effects from long-term AAS use. It is therefore important to fill in the knowledge gap on why so many abstain from seeking treatment, in order to encourage more to engage health services in the future. One important contributing factor to treatment-seeking behaviour, which is supported by this and other studies, is increased AAS knowledge among health professionals. Persons suffering serious adverse effects from long-term AAS use constitute a relatively new patient group. In the current study, we mapped differences in AAS-related health problems among those who engage health services vs. those who do not through self-reported data. However, adverse effects from AAS use might progress slowly and go unnoticed for a longer period, posing in particular a major health risk if involving cardiovascular damage. Hence, there is a need for further research investigating whether treatment seekers also differ from non-treatment seekers on objective health measures. In addition, there is need of more clinical research on harm reduction approaches for individuals with current AAS use and a desire to continue use, as well as treatment options for those with a wish to cease use. Clinicians would also benefit from increased knowledge on AAS-related health risks and how to correctly monitor this patient group with relevant examinations. In that way, harms associated with AAS use might be detected and minimized at an early phase.

## Data Availability

Not applicable.

## List of Abbreviations

AAS: Anabolic-androgenic steroids
ASIH: AAS-induced hypogonadism
AU: Alcohol unit
AUDIT: Alcohol Use Disorders Identification Test
DSM-IV: Diagnostic and Statistical Manual of Mental Disorders, 4th Edition
DUDIT: Drug Use Disorders Identification Test
GP: General practitioner
HPG: Hypothalamic–pituitary–gonadal axis
ICD-10: International Classification of Diseases 10th Revision
NOK: Norwegian krone
PCT: Post cycle therapy
REC: Regional Committees for Medical and Health Research Ethics
SCID: Structured Clinical Interview for DSM-IV
SD: Standard deviation
SUD: Substance use disorder
